# Supplementary material for: Complexome profiling of the Chlamydomonas psb28 mutant reveals TEF5 as an early PSII assembly factor
Source: Plant Cell. 2025 Mar 17;37(6):koaf055. doi: 10.1093/plcell/koaf055 (PMC12164590; doi:10.1093/plcell/koaf055)
Supplement: koaf055_Supplementary_Data [file koaf055_supplementary_data.zip › psb28_interactive.zip!/plots/intensity/qualityPlots/peptides/_Cre07.g349350.t1.2.html]

### Cre07.g349350.t1.2

Trivial name:   
SeqCoverage [median]: 38.60  
Mass: 11.34 kDa  
  
 

| Sequence | MissedCleavage | Mass [Da] | wtIntensity | muIntensity | Score |
| --- | --- | --- | --- | --- | --- |
| GGAEKYSHEEVVYGDGHHGLR | 1 | 2296.06 | 2071.46 | 0.00 | 29.28 |
| GYTYDFEHGPHYLQPEK | 0 | 2079.93 | 2008.19 | 7150.44 | 95.07 |
| KGYTYDFEHGPHYLQPEK | 1 | 2208.03 | 2308.43 | 5933.02 | 96.14 |
| YSHEEVVYGDGHHGLR | 0 | 1853.84 | 9579.79 | 6888.23 | 87.08 |
| YSHEEVVYGDGHHGLRK | 1 | 1981.94 | 897.88 | 1210.44 | 71.98 |
|  |  |  | 16865.8 | 21182.1 |  |
